# Comparative Study on the Performance of Gel Grease for High-End Equipment Based on the Synergistic Effect of Friction-Reducing Agents

**DOI:** 10.3390/gels10090573

**Published:** 2024-09-02

**Authors:** Han Peng, Yanchi Li, Linjian Shangguan, Yike Chen, Nannan Zhang

**Affiliations:** 1 School of Mechanical Engineering, North China University of Water Resources and Electric Power, Zhengzhou 450045, China; 18741466255@163.com (Y.L.); cyk08170627@163.com (Y.C.); zhangnannan@ncwu.edu.cn (N.Z.); 2 School of Water Conservancy, North China University of Water Resources and Electric Power, Zhengzhou 450046, China

**Keywords:** wear reducer, synergy, high-end equipment, gel grease

## Abstract

In the field of high-end equipment, the synergistic effect of friction-reducing agents plays an important role in the performance study of gel grease. Exploring its tribological and rheological properties can not only significantly reduce the coefficient of friction of mechanical components and enhance its viscosity at high temperatures but also effectively reduce energy consumption, thus improving the service life of high-end equipment. In this study, Schaeffler Load 460 gel grease was mixed with polysiloxane viscosity modifier (PV611) and molybdenum dialkyl dithiocarbamate (RFM3000) according to (3:1, 1:1, and 1:3), and its tribological properties and rheological properties were investigated by the MRS-10G friction and wear tester, MCR302 rotational rheometer, and crossover test. Comparative analyses of tribological and rheological properties were carried out. The results showed that the average coefficient of friction of Schaeffler Load 460 grease was reduced by 57.2%, 60%, and 71.9%, respectively, with the addition of two different ratios of friction reducers; the average diameter of abrasive spots was reduced by 44.5%, 55.4%, and 61.3%; and the shear stress and viscosity were increased by 117.94 Pa and 1295.02 mPa∙s, respectively, compared with that of the original grease, which is a good example for the lubrication of gel grease in the high-end equipment industry. This study provides a new direction and idea for the lubrication research of gel grease in the high-end equipment industry.

## 1. Introduction

High-end equipment such as aircraft engines, high-speed trains, and industrial machinery are usually operated in extreme environments, and the performance demands on the grease are extreme [1,2,3]. This equipment is often operated in harsh working environments; therefore, the requirements for lubrication materials are extremely strict [4,5]. As a common lubricating medium, gel grease can effectively reduce the friction and wear of mechanical parts, thus extending the service life of the equipment [6,7,8]. However, the performance of traditional greases under extreme working conditions is often unsatisfactory, especially at high temperatures and high pressures, where they are prone to aging and loss, which leads to a decrease in lubrication effectiveness [9,10]. Therefore, grease performance has become an important issue in the field of material science and mechanical engineering, especially regarding gel greases with excellent high-temperature performance and antioxidant properties [11,12]. Gel greases are widely used in these applications due to their good adhesion and sealing properties [13,14]. However, the requirements of these applications are not only limited to the basic performance of grease but also need to consider its stability and durability under extreme working conditions such as high temperature and high pressure [15,16]. Such research not only contributes to the progress of the industry but also effectively meets the demand for the sustainable use of high-end equipment [17,18]. Through the in-depth exploration of the synergistic mechanism of friction reducers, the design of composite friction reducers with excellent friction reduction and antiwear properties can significantly enhance the performance of grease [19,20]. Due to its unique three-dimensional network structure, gel grease is able to maintain a stable lubrication state and resist oxidation and thermal decomposition under high-temperature environments, which is of great significance for enhancing the reliability and prolonging the maintenance cycle of high-end equipment [21,22,23].

Many researchers have optimized the ratio of grease to lubricant in order to optimize the lubricating properties of grease. Bai et al. [24] investigated the addition of different ratios of gallium-based liquid metal (GBLM) in grease by four-ball test; the results showed that the extreme pressure property of the grease reaches its maximum value when the mass ratio of GBLM to a specific commercially available lithium grease reaches 1:1. Hasan Baş et al. [25] analyzed friction and wear by adding MoS_2_ and CaF_2_ at different rotational speeds, additive ratios, and contact loads, and the results showed that MoS_2_ and CaF_2_ additives could improve the wear resistance and reduce the coefficient of friction of lithium grease. Yu et al. [26] found the optimal combination of five antiwear additives in a lithium complex grease through orthogonal experimental studies. In particular, the mixture of sulfurized olefin cottonseed oil (T405), sulfurized isobutylene (SIB), and trimethylphenyl phosphate (TCP) at a ratio of 2:2:1 exhibited optimal wear resistance. In addition, T405 and TCP demonstrated synergistic effects at other ratios, further enhancing the performance of the grease. Soumya Sikdar et al. [27] investigated the addition of 0.5, 1.0, 1.5, and 2.0 wt.% graphene nanoplatelets (GNP) and 0.5, 1.0, and 1.5 wt.% hexagonal boron nitride (hBN) nanoparticles. The results show that the three nano-lubricant blends were formulated by combining GNP and hBN settings in different ratios. These blends provided the best positive synergy by reducing friction by 56% and wear by 90% compared to the base oil. M.A. Mujtaba et al. [28] investigated the performance of B30 fuel samples blended with various fuel additives. B30 fuel with DMC showed fewer wear marks, B30 fuel with nanoparticulate TiO showed the best tribological properties, and the addition of DMC and TiO combination significantly improved the engine performance. Deepak Kumar Prasad et al. [29] enhanced the tribological and thermophysical properties of lithium by adding nanomaterials. Multi-walled carbon nanotubes (MWCNT), zinc oxide (ZnO), and nano-boric acid (nBA) nanomaterials were used as additives. The coefficient of friction was analyzed in a four-ball friction and wear tester. The results showed that the surface roughness and wear of the MWCNTs hybrid grease were reduced by 59% and 84.7%, respectively, compared to the base grease. Ren et al. [30] investigated the preparation of six lithium composite greases (LCGs) using a mixture of 12-hydroxystearic acid, LiOH, and different complexing agents (azelaic acid, sebacic acid, and boric acid). The results showed that the LCGs prepared with a complexing agent combination of boric acid and sebacic acid (B/D = 1:1) with a molar ratio of 1:1 exhibited the best physicochemical and tribological properties. Liu et al. [31] synthesized two hydroxyl-functionalized ionic liquids (1-buty-3-methylimidazole salicylate (BMIM) (SA) and 1-buty-3-methylimidazole caffeate (BMIM) (CA)) as additive lubricating greases (TCGs) for titanium complexes. Tribological tests showed that both ILs improved the friction reduction and antiwear properties. Li et al. [32] used a lubricant additive package of α-zirconium phosphate (α-ZrP) and tri octyl dodecyl dioctyl phosphate (P/P). The synergistic effect of α-ZrP and P/P on the tribological properties of paraffin oil was systematically investigated. The results showed that the additives exhibited excellent friction reduction and antiwear properties. Li et al. [33] investigated the synergistic effect of 2-mercaptobenzothiazole ester-based ionic liquids and molybdenum nanoparticles in polyethylene glycol (PEG) base oils. The results showed that the hybrid additives outperformed pure PEG oil and PEG with a single additive. Muharrem Paul et al. [34] investigated the addition of different percentages of multi-walled carbon nanotubes (MWCNT) ranging from 1% to 8% to 99.9% pure copper by using powder metallurgy to evaluate their mechanical properties. The results showed that the addition of 1% MWCNT was effective at reducing wear losses and exhibited good dry lubrication, while the increase of 4% to 8% MWCNT led to an increase in wear. Ananthan D. Thampi et al. [35] formulated base oils for 16 different grease samples by mixing 12-hydroxystearic acid (12 HSA) with calcium hydroxide in different ratios from 1.25 to 2 with rice bran oil (RBO). The tribological properties of the 16 distinct grease samples, formulated through the variation of specific parameters, were evaluated utilizing a standard four-ball tester. The findings demonstrated that the alteration of these parameters during the grease formulation process had a considerable impact on the evaluated properties.

In past studies, friction reducers have been shown to be one of the effective ways to improve the performance of greases [36]. The addition of friction reducers not only significantly reduces the coefficient of friction of the lubricant but also improves its load-carrying capacity and durability [37,38]. Most of the existing studies focus on the application effect of single friction-reducing agents, and there is a lack of systematic discussion and in-depth research on the synergistic effect of different friction-reducing agents and their overall effect on grease performance [39,40]. The two friction reducers selected in this paper are polysiloxane viscosity modifier (PV611) and molybdenum dialkyl dithiocarbamate (RFM3000). PV611 has excellent friction-reducing properties, including a low coefficient of friction, high wear resistance, and good temperature stability. In addition, its good compatibility with other lubricants makes it an ideal candidate for investigating the synergistic effects of friction reducers [41]. RFM3000 has unique performance characteristics such as excellent oxidation resistance, high-temperature stability, and high extreme-pressure performance. It excels in certain applications and exhibits significant synergistic effects when combined with other friction reducers [42]. High-quality grease can form a stable and long-lasting lubrication film during the operation of the equipment through the effective proportioning and synergistic effect of friction-reducing agents, effectively reducing the direct contact of mechanical parts, thus significantly reducing friction loss and energy conversion to heat loss [43,44,45]. This not only helps to extend the service life of high-end equipment and reduce the mechanical failure rate but also reduces energy consumption [46,47].

In this study, Schaeffler Load 460 gel grease was mixed with polysiloxane viscosity modifier (PV611) and molybdenum dialkyl dithiocarbamate (RFM3000) in accordance with (3:1, 1:1, and 1:3), respectively, and the systematic performance evaluation of the greases with different ratios was carried out by the combined experiments of the friction and wear tester and the rheometer, and the MRS-10G friction and wear tester. The systematic performance evaluation of the greases was carried out by the MRS-10G Friction and Wear Tester. The test results not only quantify the friction coefficient and wear characteristics of each grease but also provide experimental data support for the further optimization of gel grease formulations. The MCR rotational rheometer, with accurate rheological testing, can analyze the trend of grease at 80 °C, along with shear rate and shear stress, and assess its influence on the friction performance as well as rheological characteristics of the grease [48]. This study explores the interaction between friction-reducing agents in gel greases by systematically investigating their synergistic effects to optimize lubrication performance. Focusing on the field of high-end equipment, the study investigates how to improve the performance of grease as well as its important practical application value. This thesis proposes improved gel-like grease formulations, combining theoretical analysis and experimental validation to deeply understand and optimize the mechanism of friction-reducing agents. These innovative studies have a positive impact on the development of high-end equipment grease technology. It is expected that the experimental results of this study will provide a scientific basis for optimizing the formulation design of gel grease for high-end equipment and promote the progress of lubrication technology in the application of extreme working conditions, so as to improve the reliability and service life of high-end equipment. In addition to improving the performance and longevity of high-end equipment, optimizing gel grease formulations plays an important role in addressing global environmental challenges. Developing more efficient lubricants directly contributes to waste reduction and minimizes the environmental footprint associated with the disposal and frequent replacement of industrial lubricants. By extending the life cycle of lubricants and equipment, this research is in line with global sustainability goals aimed at reducing industrial waste and conserving energy [49]. In addition, the effective use of friction reducers in grease formulations can significantly reduce energy consumption, further supporting efforts to mitigate the environmental impact of industrial operations. These advances in lubrication technology not only improve efficiency but also promote responsible resource management in line with current environmental priorities [50].

## 2. Results and Discussion

### 2.1. Tribological Performance Test

#### 2.1.1. Tribological Properties of Schaeffler Load 460

**Figure 1:**
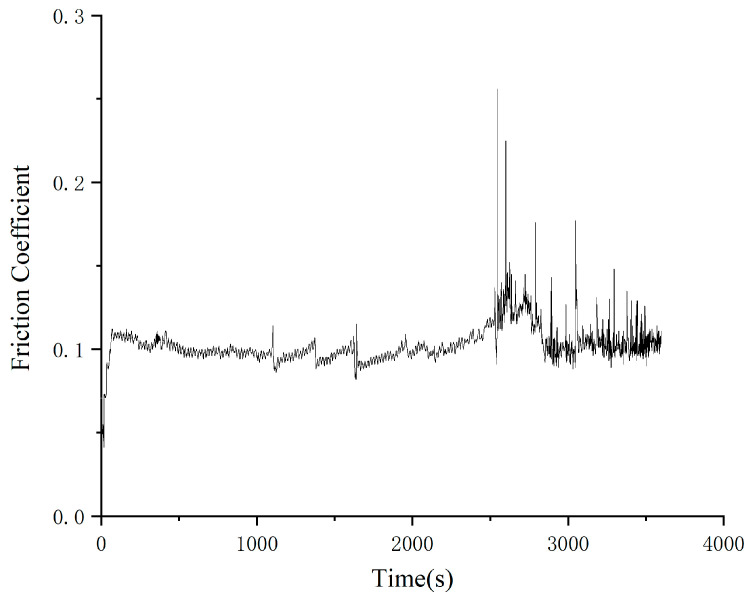
Friction coefficient-time curve for Load 460 grease.

Experimental friction and wear studies were carried out on Schaeffler Load 460 gel grease without a wear reducer; the coefficient of friction-time curve is shown in Figure 1.

**Figure 2:**
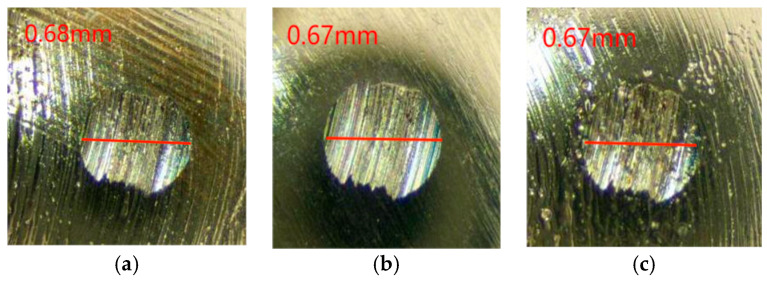
Wear spot diameter of load 460 grease, (**a**) 1 ball abrasion spot, (**b**) 2 ball abrasion spots, (**c**) 3 ball abrasion spots.

As can be seen from the change rule of the friction coefficient-time curve, the friction coefficient of Schaeffler Load 460 gel grease without additives fluctuated greatly during the experiment. The coefficient of friction reaches a relatively stable state at 160 s and starts to fluctuate greatly at 2550 s. The minimum value of the coefficient of friction was 0.082, the maximum value was 0.257, and the average coefficient of friction was 0.11. The wear spots of the steel balls of Schaeffler Load 460 grease are shown in Figure 2. The diameters of the wear spots were 0.68 mm, 0.67 mm, and 0.67 mm in order after observation by microscope, and the average diameter of the wear spots was 0.673 mm.

As can be seen from Figure 1 and Figure 2, Schaeffler Load 460 gel grease is widely used in the field of high-end equipment, but its friction and wear properties are not ideal. The initial fluctuation of the experiment is due to the imperfect contact of the friction surface and the process of lubrication film formation. When the experiment was carried out for 160 s, the friction coefficient reached a relatively stable state, which could be attributed to the formation of a relatively uniform lubricant film on the friction surface, reducing the contact and friction between the friction surfaces. As time passed, the grease showed extremely unstable behavior in terms of coefficient of friction. This is due to the fact that Schaeffler Load 460 gel grease lacks effective additives during friction, resulting in poor lubrication properties. The lubricant film is damaged by external factors such as high temperature, pressure, and shear. The inhomogeneity of the microstructure and chemical composition of the friction surface leads to localized frictional failures during the friction process. This failure leads to an instantaneous increase or decrease in the coefficient of friction, which makes the surface of the friction pair unable to form a stable lubrication film, and the friction surface is easy to wear out by direct contact, which results in a wide range of overall changes in the coefficient of friction. In addition, the observation of steel ball wear spots shows the wear of the surfaces during the friction process. Wear is caused by the contact and relative motion between the friction surfaces, and the wear process changes the morphology and chemical composition of the friction surfaces, which in turn affects the stability of the friction properties. The fluctuations of the friction coefficient-time curves and the observed results of the steel ball wear spots reflect the complex interactions between the grease and the friction surfaces during the friction process; therefore, further research on the composition and additives of the grease and strict control of the experimental conditions are needed to ensure the stability and reliability of the friction performance.

#### 2.1.2. Effect of PV611 on Tribological Properties of Greases

**Figure 3:**
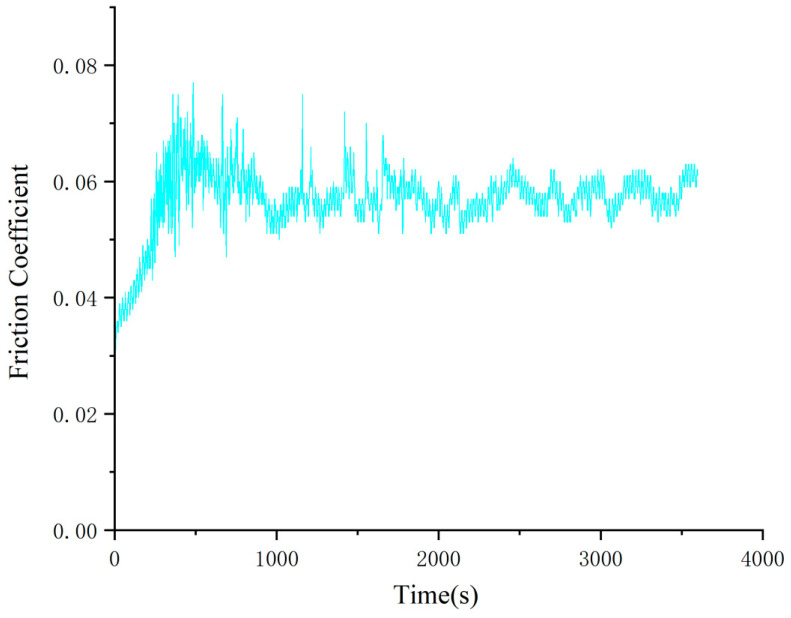
Friction coefficient-time curve of grease with wear-reducing agent PV611.

Friction wear experimental studies were carried out on Schaeffler Load 460 gel grease with the addition of the wear reducer PV611, and the coefficient of friction-time curve is shown in Figure 3.

**Figure 4:**
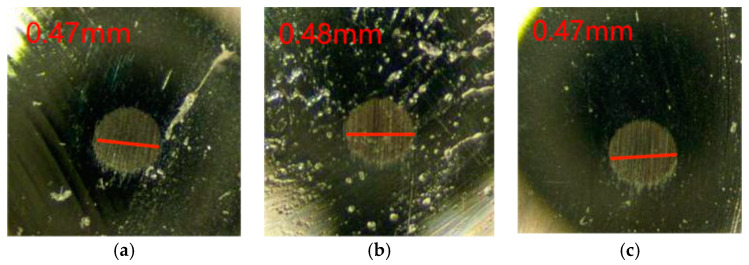
Spot diameter of grease with wear-reducing agent PV611. (**a**) 1 ball abrasion spot, (**b**) 2 ball abrasion spots, (**c**) 3 ball abrasion spots.

From the change rule of the graph line in Figure 3, it can be seen that the friction coefficient of Schaeffler Load 460 gel grease with the additive of wear-reducing agent PV611 fluctuates a lot before 1900 s, and it stabilizes at about 0.055 at 2000 s. During the experiment after the friction coefficient was stabilized, the minimum value of the friction coefficient was 0.052, the maximum value was 0.063, and the experimental data showed that the average friction coefficient was 0.061. The wear spots of the steel balls of the Schaeffler Load 460 gel grease with the additive PV611 were observed (as shown in Figure 4). The diameters of the wear spots were 0.47 mm, 0.48 mm, and 0.47 mm in order after observation by microscope, and the average diameter of the wear spots was 0.473 mm.

As can be seen from Figure 3 and Figure 4, in terms of friction coefficient, the experimental data show that the average friction coefficient of Schaeffler Load 460 gel grease in steady state is 0.061, which is significantly reduced compared with the original grease. This difference may be due to the special components in the wear reducer PV611, which can form a stable lubricant film and a smoother interface on the friction surface, thus reducing the frictional resistance and lowering the coefficient of friction, but its film-forming time is longer, and the friction coefficient of Schaeffler Load 460 gel grease is 0.061 in the steady state; this can result in equipment experiencing higher friction and wear during start-up or early operation, which can exacerbate component damage, increase start-up resistance, delay the effectiveness of lubrication, and lead to increased heat. In addition, the failure to form a good lubricant film can lead to corrosion risks, increased vibration, and noise. The selection of wear reducers with rapid film formation properties, as well as proper application and maintenance management, are key measures to avoid these problems. Therefore, for high-end equipment, if the grease film formation time is too long, the friction may increase when the equipment is started up because the grease has not yet fully lubricated the surfaces and the direct contact between the friction surfaces may lead to additional wear. In addition, the wear reducer PV611 consists mainly of siloxane chains, and the length and structure of these chains can affect viscosity and lubrication properties. Inappropriate siloxane chains may lead to grease failure at high temperatures or high pressures, thus affecting the lubrication of main bearings and other related parts of high-end equipment.

#### 2.1.3. Effect of RFM3000 on Tribologs of Greases

**Figure 5:**
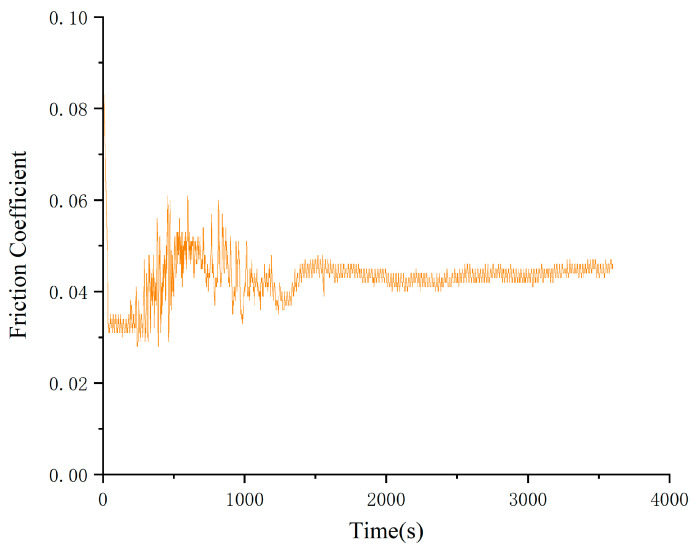
Friction coefficient-time curve of grease with wear-reducing agent RFM3000.

Friction wear experimental studies were carried out on Schaeffler Load 460 gel grease with the addition of wear reducer RFM3000; the coefficient of friction-time curve is shown in Figure 5.

**Figure 6:**
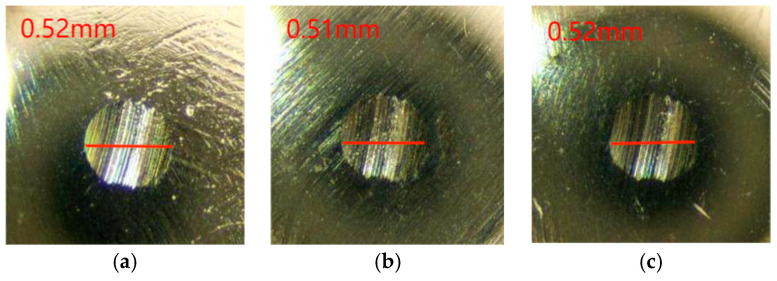
Spot diameter of grease with wear-reducing agent RFM3000. (**a**) 1 ball abrasion spot, (**b**) 2 ball abrasion spots, (**c**) 3 ball abrasion spots.

The coefficient of friction of Schaeffler Load 460 gel grease with wear reducer RFM3000 shown in Figure 5 fluctuates considerably up to 1250 s and then levels off. At around 1500 s, the friction coefficient reaches a relatively stable state. The minimum value of the coefficient of friction was 0.04, the maximum value was 0.049, and the average coefficient of friction was 0.045. The wear spots of the steel balls of the RFM3000-added Schaeffler Load 460 Gel Grease are shown in Figure 6. The diameters of the wear spots were 0.52 mm, 0.51 mm, and 0.52 mm in order after observation by microscope, and the average diameter of the wear spots was 0.516 mm.

As can be seen in Figure 5 and Figure 6, the stability of the coefficient of friction-time curve of Schaeffler Load 460 gel grease with the addition of the wear reducer RFM3000 is significantly improved. The large fluctuations in the coefficient of friction at the initial stage of the experiment are either due to the adaptation and compatibility between the grease or the contact surface being adjusted. The grease may require an adaptation period during its initial use in order to form a homogeneous lubricant film inside the bearing. During this process, the fluidity and distribution of the grease may change, resulting in fluctuations in the coefficient of friction. Schaeffler Load 460 gel grease with the addition of the wear reducer RFM3000 contains new chemical components, which exhibit different rheological properties when the temperature is raised, which also leads to instability in the coefficient of friction, and 1500 s later, the coefficient of friction stabilizes, which is the result of the synergistic effect of MoDTC in the instrumented shear and the high degree of formation of the lubricant film. For high-end equipment, the stability of the coefficient of friction is crucial.

#### 2.1.4. Effect of Mixing PV611 with RFM3000 (3:1) on the Tribological Properties of Greases

**Figure 7:**
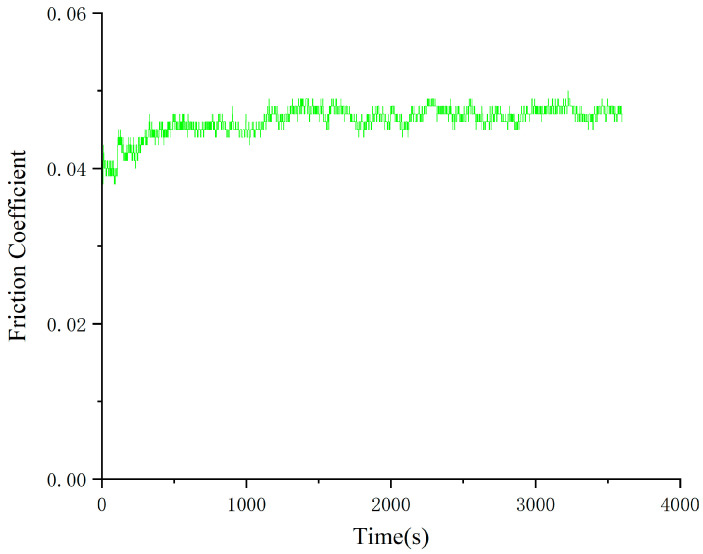
Friction coefficient-time curve of grease added with PV611 mixed with RFM3000 (3:1).

Friction and wear experimental studies were carried out on Schaeffler Load 460 gel grease mixed with the wear reducer PV611 and RFM3000 (3:1), and the coefficient of friction-time curve is shown in Figure 7.

**Figure 8:**
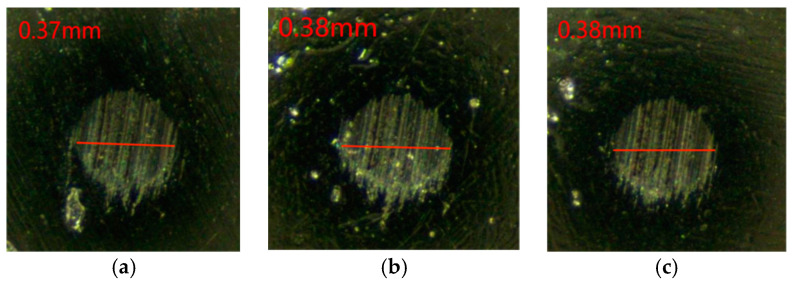
Diameter of wear spots with the addition of grease mixed with PV611 and RFM3000 (3:1). (**a**) 1 ball abrasion spot, (**b**) 2 ball abrasion spots, (**c**) 3 ball abrasion spots.

As can be seen from the change rule of the graph line in Figure 7, the friction coefficient of Schaeffler Load 460 gel grease mixed with PV611 and RFM3000 (3:1) remained stable during the experiment and the total friction coefficient was relatively small. The coefficient of friction reached a relatively stable state at 200 s. After the friction coefficient was stabilized, the minimum value of the friction coefficient was 0.039, the maximum value was 0.049, and the average friction coefficient was 0.047. The grinding spots of steel balls with Schaeffler Load 460 gel grease mixed with PV611 and RFM3000 (3:1) are shown in Figure 8. The grinding spot diameters were 0.37 mm, 0.38 mm, and 0.38 mm, in that order, after observation by microscope, and the average grinding spot diameter was 0.373 mm.

As can be seen in Figure 7 and Figure 8, the stability of the coefficient of friction of Schaeffler Load 460 gel grease using a mixture of PV611 and RFM3000 (3:1) has been significantly improved. The problem of unstable friction coefficient in the early stage of the experiment has been significantly improved. RFM3000 contains MoDTC, which is an important organic molybdenum compound. This compound has a special structure in which two sulfur atoms form a coordination bond with the molybdenum atom, and the carbamate group forms a coordination bond with the molybdenum atom through the nitrogen atom, which is able to show good extreme-pressure antiwear performance in the base oil. It improves the formation and maintenance of the lubricant film when used in conjunction with PV611, thereby increasing the stability of the coefficient of friction. For high-end equipment, where the stability of the coefficient of friction is critical, this combination not only improves the stability of the coefficient of friction but also improves the quality of the lubricant film, which extends the life of the equipment and reduces maintenance costs.

#### 2.1.5. Effect of Mixing PV611 with RFM3000 (1:1) on the Tribological Properties of Greases

**Figure 9:**
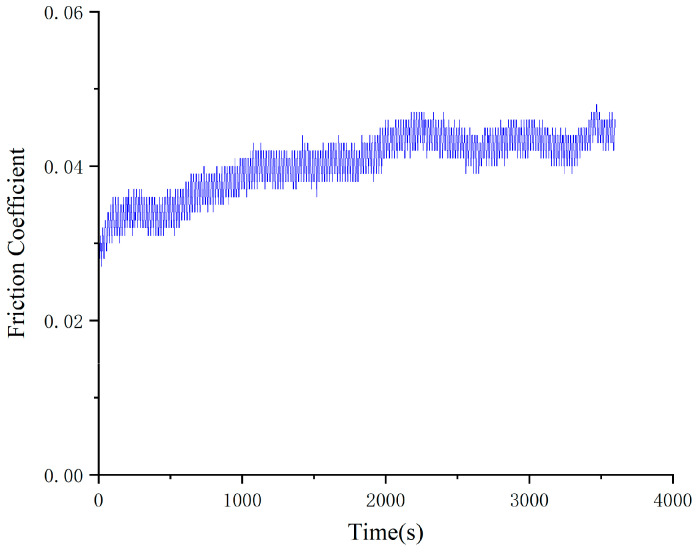
Friction coefficient-time curve of grease added with PV611 mixed with RFM3000 (1:1).

Friction and wear experimental studies were carried out on Schaeffler Load 460 gel grease mixed with the wear reducer PV611 and RFM3000 (1:1), and the coefficient of friction-time curve is shown in Figure 9.

**Figure 10:**
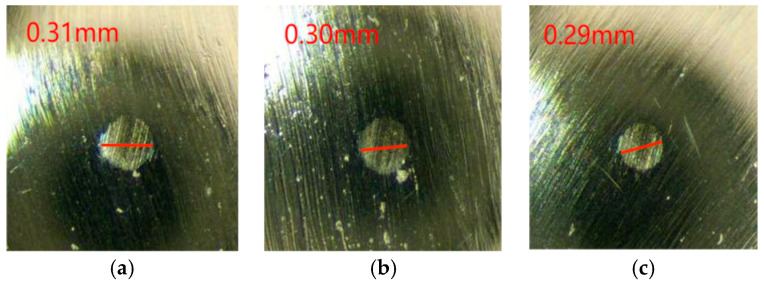
Diameter of wear spots with the addition of grease mixed with PV611 and RFM3000 (1:1). (**a**) 1 ball abrasion spot, (**b**) 2 ball abrasion spots, (**c**) 3 ball abrasion spots.

As can be seen from the change rule of the graph line in Figure 9, the friction coefficient of Schaeffler Load 460 gel grease mixed with PV611 and RFM3000 (1:1) remained stable during the experimental process, and the total friction coefficient had an overall upward trend. The minimum value of the coefficient of friction was 0.027, the maximum value was 0.047, and the average coefficient of friction was 0.044. The wear spots of the steel balls of Schaeffler Load 460 gel grease mixed with PV611 and RFM3000 (1:1) are shown in Figure 10. The grinding spot diameters were 0.31 mm, 0.30 mm, and 0.29 mm, in that order, after observation by microscope, and the average grinding spot diameter was 0.30 mm.

As can be seen from Figure 9 and Figure 10, the coefficient of friction of Schaeffler Load 460 gel grease mixed with PV611 and RFM3000 (1:1) is lower than that of the grease mixed with (3:1), and with the addition of RFM3000, the protective film formed by MoDTC on the friction surface can effectively isolate the metal surface and reduce the direct metal-to-metal contact, thus reducing the generation of particles caused by friction and lowering the coefficient of friction. With the addition of RFM3000, the protective film formed by MoDTC on the friction surface can effectively isolate the metal surface and reduce the direct metal-to-metal contact, thus reducing the generation of particles caused by friction and lowering the friction coefficient. Under high temperatures and high-speed operation, the polysiloxane in PV611 can adjust the viscosity of the grease as needed, and increasing the viscosity appropriately can prevent the loss of grease, maintain the lubrication effect, and ensure that proper lubrication can be provided under different working conditions. The diameter of the wear spot also becomes smaller due to the reduction of particle generation and viscosity adjustment of the additives. However, its coefficient of friction is still increasing with time, which is because, at this ratio, the composition of the two wear reducers is close to equal, leading to an increase in the interaction between the different components, changing the formation and nature of the lubricating film and leading to changes in the coefficient of friction. Therefore, further changes in their ratios are required.

#### 2.1.6. Effect of Mixing PV611 with RFM3000 (1:3) on the Tribological Properties of Greases

**Figure 11:**
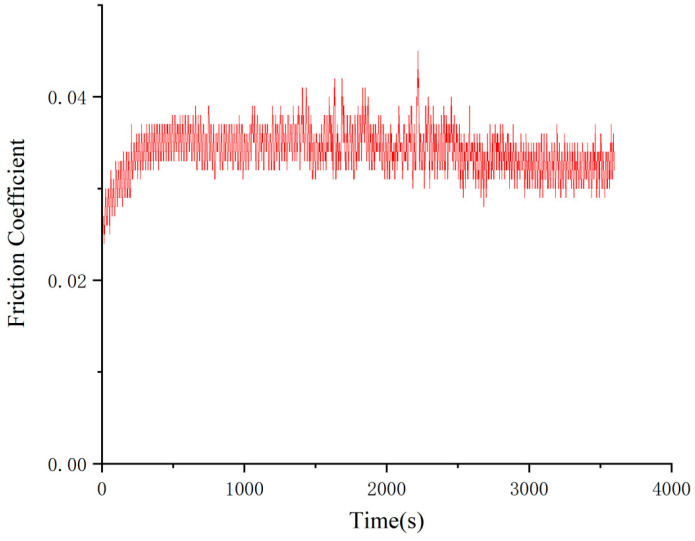
Friction coefficient-time curve of grease added with PV611 mixed with RFM3000 (1:3).

Friction and wear experimental studies were carried out on Schaeffler Load 460 gel grease mixed with the wear reducer PV611 and RFM3000 (1:3), and the coefficient of friction-time curve is shown in Figure 11.

**Figure 12:**
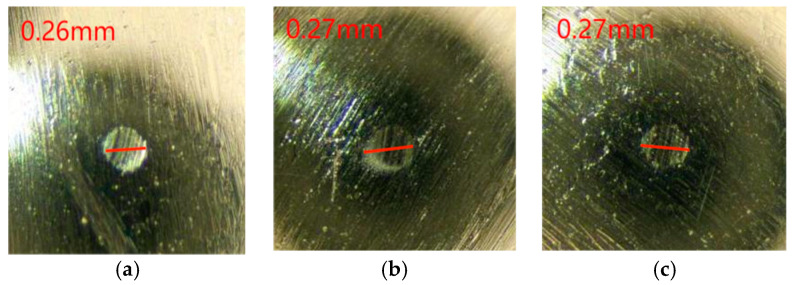
Diameter of wear spots with the addition of grease mixed with PV611 and RFM3000 (1:3). (**a**) 1 ball abrasion spot, (**b**) 2 ball abrasion spots, (**c**) 3 ball abrasion spots.

As can be seen from the change rule of the graph line in Figure 11, the friction coefficient of Schaeffler Load 460 gel grease mixed with PV611 and RFM3000 (1:3) remained stable during the experimental process, and the total friction coefficient had an overall decreasing trend. The minimum value of the coefficient of friction was 0.027, the maximum value was 0.045, and the average coefficient of friction of the experimental data was 0.032. The wear spots of the steel balls of Schaeffler Load 460 grease mixed with PV611 and RFM3000 (1:3) were added (as shown in Figure 12). The grinding spot diameters were 0.26 mm, 0.27 mm, and 0.27 mm, in that order, after observation by microscope, and the average grinding spot diameter was 0.266 mm.

As can be seen in Figure 11 and Figure 12, Schaeffler Load 460 gel grease with PV611 mixed with RFM3000 (1:3) has a lower average coefficient of friction and smaller wear spot diameter than the grease with the first two additives, due to the fact that it relies on MoDTC, a component of RFM3000, to protect the lubricant from oxidation and degradation during friction operations. This is due to the fact that the grease relies more on the MoDTC component contained in RFM3000, which breaks down into molybdenum disulphide nanoparticles and diethylhexylcarbamate as the temperature rises during friction operations, effectively protecting the lubricant from oxidation and degradation and reducing the breakdown of solid particles from the friction surfaces at elevated temperatures to reduce the burden on the equipment operation. Comparatively speaking, the dependence of the grease on pv611 is not as high as that of RFM3000, but the quaternary ammonium groups, siloxane blocks, polyalkylene oxide structural units, and terminal ester groups contained in PV611 can play a role in regulating the viscosity of the grease and preventing it from being destroyed at high temperatures. The combination of PV611 and RFM3000 creates a better overall lubricant system through the low-friction properties of the silicone chains and the extreme-pressure properties of the molybdenum compounds. PV611 provides excellent viscosity stability and low friction, while RFM3000 enhances antiwear and extreme-pressure properties, and the complementary chemistry of the two significantly improves the overall performance of the lubricant. PV611 gel grease blended with RFM3000 (1:3) is a synergistic blend of two additives that can be used in high-end equipment to enhance performance, extend equipment life, and ensure reliable operation under extreme operating conditions.

#### 2.1.7. Comparative Analysis of Tribological Experiments

Previous studies have typically focused on evaluating the performance of a single friction reducer, such as the respective coefficient of friction, wear rate, and high temperature resistance of PV611 or RFM3000. These studies provide basic data on the performance characteristics of various types of friction reducers but usually do not delve into the synergistic effects between different friction reducers. This study reveals the synergistic effect of PV611 and RFM3000 when used together, significantly improving friction performance and wear resistance. This synergistic effect not only reduces the coefficient of friction but also prolongs the lubricant’s service life and exhibits higher temperature stability and oxidation resistance compared to the results of previous individual studies. These new findings suggest that combining these two friction reducers can enhance lubrication under a wider range of application conditions.

**Figure 13:**
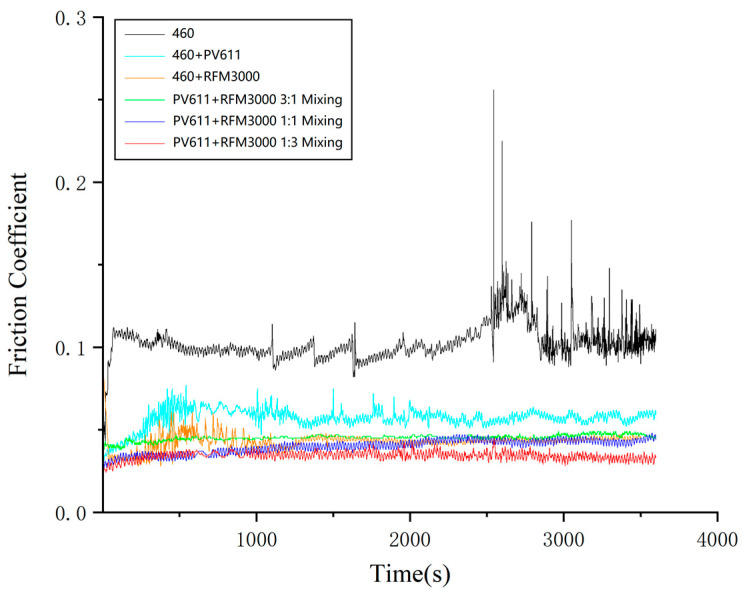
Friction coefficient-time curves of basic grease and experimental grease.

**Figure 14:**
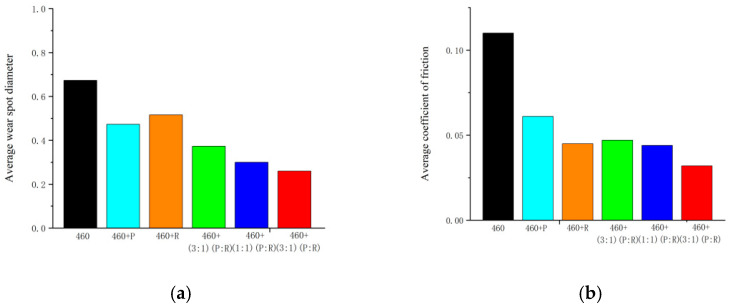
Average coefficient of friction and average spot diameter of base grease and experimental grease. (**a**) Average coefficient of friction, (**b**) Average spot diameter.

Figure 13 and Figure 14 show the results of a series of friction and wear experiments involving the original gel grease, two different wear reducers, and samples of them mixed in different ratios (3:1, 1:1, and 1:3). The graphs clearly show the comparison between the original gel grease and the experimental grease in terms of friction coefficient over time. By comparing the average coefficient of friction and the average diameter of wear marks, the effect of different ratios of wear-reducing agents on the friction and wear performance of the gel grease under synergistic action is more clearly revealed. The results show that Load 460 gel grease with PV611 mixed with RFM3000 (1:3 ratio) exhibits the lowest average coefficient of friction and average wear diameter.

From the results of this experiment, both the wear reducer PV611 and RFM3000 can reduce the average coefficient of friction and the average spot diameter of the gel grease. The synergistic effect of the two agents through a certain proportion can change the lubrication performance of the gel grease better. Mixing PV611 and RFM3000 in a ratio of (1:3) and applying them to Schaeffler Load 460 gel grease can better utilize the synergistic effect of the two additives and give full play to the performance advantages of each additive. For high-end equipment, it can ensure that the equipment maintains a good working condition under high load and high speed, thus improving productivity. Secondly, due to its excellent wear and corrosion resistance, it can reduce the wear and failure rate of equipment and reduce the frequency of maintenance. The long-term stability of this grease results in fewer grease changes and further cost savings.

This study has focused on specific types of high-end equipment, such as high-temperature or high-load applications, but has not adequately considered the effects of different operating conditions (e.g., load, speed, and temperature range) on grease performance. Differences in the needs of different equipment may lead to significant differences in grease effectiveness. Future research should extend to a wide range of equipment types to evaluate the performance of greases PV611 and RFM3000 in a variety of equipment, especially under extreme operating conditions. At the same time, environmental factors such as humidity and contaminants need to be taken into account, long-term performance and ageing tests need to be carried out to fully understand their long-term stability, and the compatibility of the grease with the equipment materials needs to be thoroughly investigated to avoid possible chemical reactions or corrosion problems, thus ensuring the grease’s reliability in a wide range of real-world application environments.

### 2.2. Rheological Property Test

#### 2.2.1. Comparative Analysis of Shear Stress

**Figure 15:**
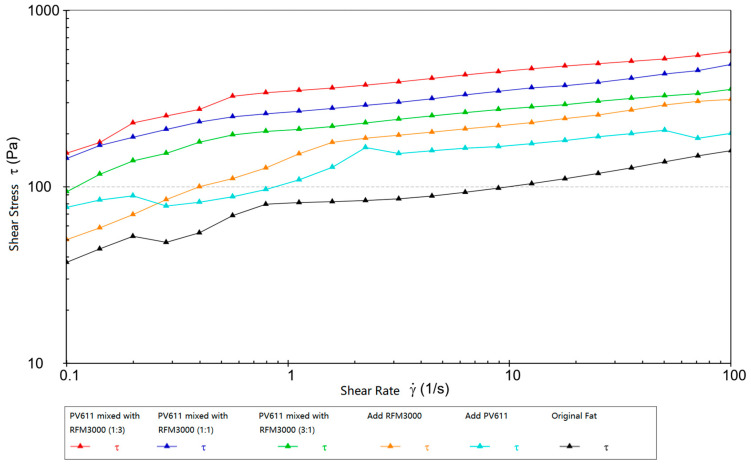
Shear stress-shear rate curves of basic grease and experimental grease.

Experimental rheological studies were carried out on Schaeffler Load 460 gel grease and test grease with added wear reducer, with shear stress-shear rate curves (shown in Figure 15).

Typically, high temperatures cause grease to run off or become thin, thus reducing its lubricating effect. By using grease with a high-shear stress, it is possible to maintain more consistent lubrication at high temperatures. The high-shear stress helps grease form a longer-lasting and more stable lubricant film, which reduces friction and wear. In high-temperature environments, equipment stability and reliability are critical to productivity. High-shear stress grease can maintain a stable lubrication condition of the equipment, reduce the negative impact of lubrication changes, and ensure the stable operation of high-end equipment for a long time.

As shown in Figure 15 when the wear reducer PV611 is mixed with RFM3000 (1:3) and added to Schaeffler Load 460 gel grease compared with the original grease, the initial shear stress is increased from 37.34 pa to 155.28 pa, which is obviously higher at high temperatures. This is because the wear reducer PV611 can form an effective lubricant film under a high-temperature environment and reduce the loss of lubricant under high-shear stress conditions, thus improving the shear stress performance of the grease. Its molecular structure enables it to stabilize the viscosity when added to grease and to maintain its lubricating properties under different operating conditions. The wear reducer RFM3000 is able to form a chemically reactive film between the grease coating and the metal surface, effectively reducing friction and wear when metal surfaces come into contact. The addition of RFM3000 improves the load-carrying capacity and wear resistance of the grease under high pressure, resulting in better performance under high-shear stress conditions. PV611 and RFM3000 can be used in Schaeffler Load 460 gel grease in a (1:3) ratio for better synergy.

#### 2.2.2. Comparative Viscosity Analysis

**Figure 16:**
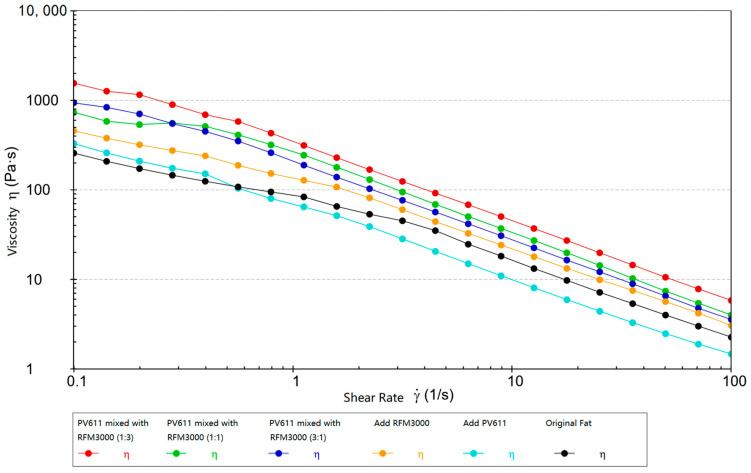
Viscosity-shear rate curves of basic grease and experimental grease.

Experimental rheological studies were carried out on Schaeffler Load 460 gel grease and test grease with added wear reducer, and the viscosity-shear rate curves are shown in Figure 16.

Usually, under a high-temperature environment, it makes the general lubricant decompose or oxidize quickly; it thus loses its lubricating performance and even forms a gel-like substance. The viscosity of the grease, if it is too low, may lead to the rapid loss of lubricant under high-speed movement or a heavy load, which greatly reduces the lubricating effect and hinders the normal functioning of the mechanical parts. In contrast, greases with high viscosity usually contain antioxidants and high-temperature stabilizers, which are able to resist oxidation and decomposition in high-temperature environments, maintain the durability and stability of their lubrication function, adhere better to metal surfaces, and are less susceptible to high-temperature loss, thus maintaining stable lubrication effects. This means that equipment can be down for maintenance less often, reducing production interruptions and maintenance costs. For high-end equipment, this not only improves reliability and stability but also reduces overall operating costs.

As shown in Figure 16 when the wear reducer PV611 is mixed with RFM3000 (1:3) and added to Schaeffler Load 460 gel grease compared with the original grease, the initial viscosity is increased from 257.28 mPa-s to 1552.3 mPa-s, which is obviously higher viscosity at high temperatures. This is due to the fact that the wear reducer PV611 can significantly increase the viscosity index of the grease, and the viscosity of the grease changes less even at different temperatures. This property is particularly important in situations where operating temperatures fluctuate as it ensures that the grease does not thin excessively at high temperatures or thicken excessively at low temperatures, thus maintaining stable lubricating properties. Because polysiloxane improves the fluidity and lubricity of the grease, it maintains its viscosity during the lubricant’s operating life, reducing grease degradation over extended periods of time. The film layer formed by the wear reducer RFM3000 in the lubricant also effectively reduces the coefficient of friction and reduces the friction and wear of metal parts in motion contact, thus extending the service life of the grease and the mechanical parts. The advantages of each of the two products can be fully utilized through synergistic action, thus enhancing the viscosity and lubrication performance of the grease in an integrated manner. The PV611 improves the viscosity index and viscosity stability, while RFM3000 enhances extreme pressure and anti-wear capability. PV611 improves viscosity index and viscosity stability, while RFM3000 enhances extreme pressure and wear resistance, combining them to cope with more complex operating conditions. The (1:3) mix ratio usually balances the needs of the grease under different operating conditions, such as high loads, high temperatures, or variable temperatures, to maintain consistent lubrication and performance. The optimization of grease viscosity and shear stress interactions can significantly improve grease performance, including enhancing the durability and stability of lubrication, as well as its reliability and efficiency under high loads, high speeds, and high temperatures. This is important for protecting high-end equipment, extending service life, and reducing maintenance costs.

## 3. Conclusions

### 3.1. Conclusions

In this paper, using the MRS-10G friction and wear tester and the MCR rotational rheometer, the tribological performance and rheological experiments were carried out on high-end equipment gel grease Schaeffler Load 460 added with two additives, PV611 and RFM3000, which were mixed according to (3:1, 1:1, and 1:3). The friction coefficient-time curve as well as wear spot diameter were measured under the synergistic action of the grease with different proportions of wear-reducing agents, and their average friction coefficient and average wear spot diameter were calculated, and their changes in shear stress and viscosity; the following conclusions were drawn:

(1) The average coefficient of friction of the experimental grease Schaeffler Load 460 was 0.11; with the addition of PV611, the coefficient of friction was reduced to 0.061; with the further addition of RFM3000, the coefficient of friction was further reduced to 0.045. The coefficients of friction for the different mixing ratios are shown as follows: the experimental grease with the addition of PV611 mixed with RFM3000 (3:1) was 0.047; the experimental grease with the (1:1) mixture was 0.044; and the experimental grease with the (1:3) mixture was the lowest at 0.032. These results show that the experimental grease using PV611 mixed with RFM3000 (1:3) significantly reduces the coefficient of friction in all test conditions.
(2) In friction wear experiments, it was observed that the average wear spot diameter of the experimental grease for Schaeffler Load 460 was 0.673; with the addition of PV611, the wear spot diameter was reduced to 0.473; and with the further addition of RFM3000, the wear spot diameter was reduced to 0.516. The experimental results at different mixing ratios showed that the diameter of the abraded spot of the experimental grease with the addition of PV611 mixed with RFM3000 according to (3:1) was 0.373; the diameter of the abraded spot mixed according to (1:1) was 0.3; and the diameter of the abraded spot mixed according to (1:3) was the smallest, which was 0.266. These results show that the use of experimental grease mixed with PV611 and RFM3000 (1:3) was able to significantly reduce the diameter of the abrasive spots in all test conditions.
(3) In the process of a shear rate increase from 0/s to 100/s, the experimental grease using PV611 mixed with RFM3000 (1:3) shows more obvious viscosity characteristics and greater shear stress, which makes it possible to maintain better viscosity characteristics in a high-temperature environment.

The experimental results showed that the greases with the addition of PV611 and RFM3000 exhibited significant improvements in both the coefficient of friction and the degree of wear. Specifically, the coefficient of friction was reduced by 57.2%, 60%, and 71.9%, while the wear spot diameter was reduced by 44.5%, 55.4%, and 61.3%, respectively. These improvements are beyond the typical range available. While previous studies have found that greases with a single friction reducer typically show reductions in the coefficient of friction in the range of 30–50%, the significant improvements in this study indicate that the synergistic action of PV611 and RFM3000 can significantly optimize lubrication performance. The significant increase in shear stress and viscosity (117.94 Pa and 1295.02 mPa-s, respectively) suggests that the addition of friction reducers improves the fluidity and load carrying capacity of the grease. In contrast to previous studies, which reported grease viscosity enhancement in the range of 500–1000 mPa-s, the enhancement in this study was more significant, suggesting a strong synergistic effect of the friction reducer combination on the rheological properties. The synergistic effect of PV611 and RFM3000 significantly improves the overall performance of the grease. PV611 increases the viscosity of the grease and enhances the stability of the lubricant film and load carrying capacity. The combination of RFM3000, a solid lubricant that reduces friction and wear, optimizes the performance of the grease under extreme conditions. This study validates the superior performance of the combined friction reducer compared to previous studies that used only a single type of friction reducer.

### 3.2. Future Perspectives

This study not only verifies the importance of the synergistic effect of PV611 and RFM3000 in greases but also provides a new direction for the future development of greases for high-end equipment. Future research can further explore more combinations and optimization of friction-reducing agents, especially under more complex working conditions, to enhance the operational efficiency and reliability of high-end equipment.

(1) The tribological performance and rheological properties of the grease can be further optimized by continuously adjusting the mixing ratio of PV611 and RFM3000. In particular, it was found that the grease mixed with PV611 and RFM3000 (1:3) had the best performance in terms of reducing the coefficient of friction and the diameter of the wear spots in the experiments, and future studies can explore more precise mixing ratios to obtain superior performance.
(2) The grease mixed with PV611 and RFM3000 (1:3) shows good viscosity characteristics and shear stress at high shear rates and high temperatures, which is especially important for high-end equipment such as bearings and gearboxes at high loads and high speeds. Future research can further verify its stability and durability under different temperature and pressure conditions to ensure its wide application in industrial and high-end equipment.

## 4. Materials and Methods

### 4.1. Laboratory Equipment and Materials

#### 4.1.1. MRS-10G Friction and Wear Tester

**Figure 17:**
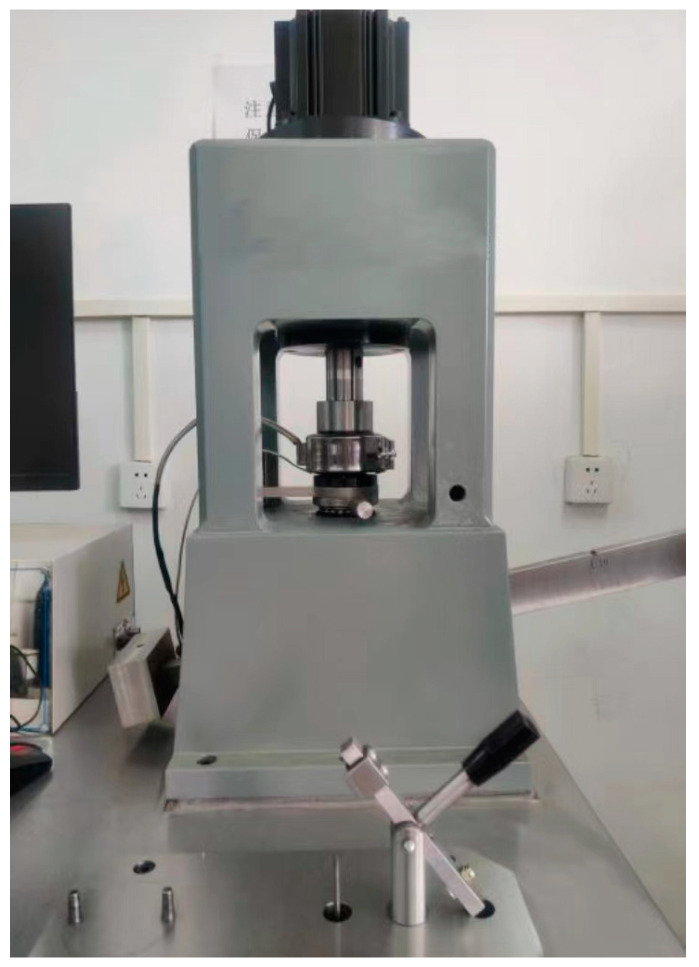
MRS-10G friction and wear tester.

The tribological properties of gel grease can be fully evaluated using the MRS-10G Friction and Wear Tester (shown in Figure 17). This machine is capable of simulating real working conditions, such as by adjusting the spindle speed (200 to 3000 r/min) and axial loading (98 to 9800 N), and by heating the oil cup to 250 °C. The spindle speed and axial loading can be adjusted to simulate friction and wear under real working conditions, providing a reliable simulation environment. They are capable of accurately measuring the friction force and coefficient of friction of grease or other materials under different working conditions to evaluate the friction performance of materials. Through the accurate measurement of wear amount and wear morphology, an in-depth study of the wear resistance and wear mechanism of materials can be undertaken. Through the experimental data, we can understand the difference in the tribological performance of different gel greases under actual working conditions, which provides an important reference for the selection of greases and wear reducers.

#### 4.1.2. MCR Rotational Rheometer

**Figure 18:**
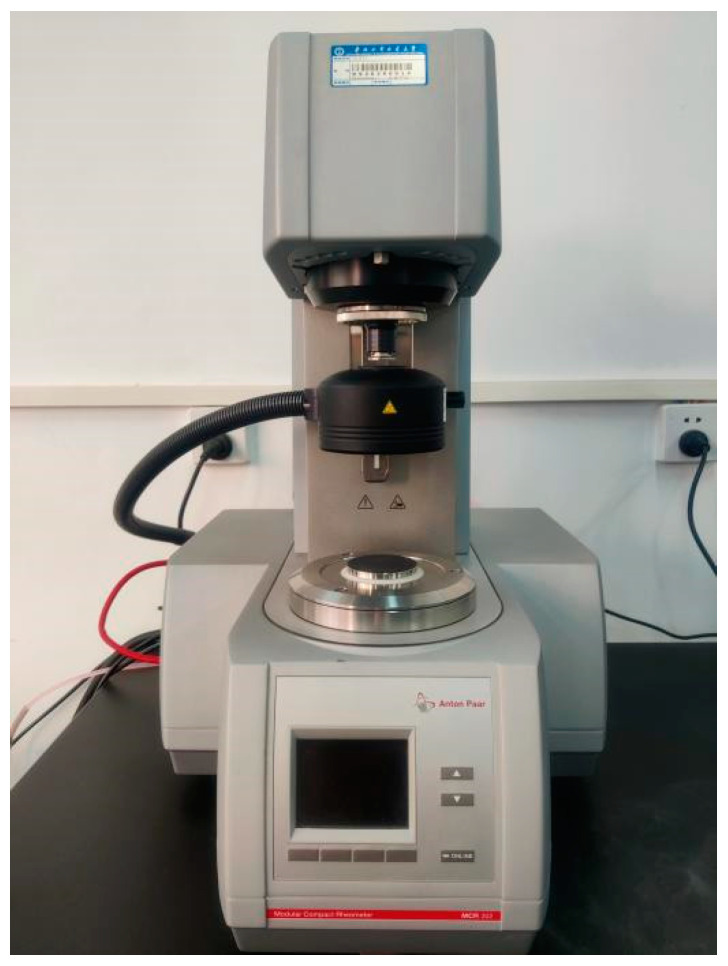
MCR rotational rheomete.

The rheological properties of gel greases can be comprehensively evaluated using the MCR rotational rheometer (shown in Figure 18). The instrument can perform rheological performance tests over a wide measuring range, with a rotational speed range of 10^−6^ to 200 r/min, a measuring torque of 10 μN·m to 0.2 N·m, and a normal force of 0.001 N to 50 N. The instrument is capable of accurately measuring the viscosity of a substance at different shear rates, including rheological behaviors such as shear dilution, shear thickening, and shear thinning. It can measure the stress response of substances under the action of applied shear force, including the relationship between shear force and shear rate. A variety of operating modes and testing options are available to adapt to the testing needs of different samples. The experimental data can reveal the rheological performance changes of different gel greases under actual working conditions, providing a scientific basis for the selection of suitable gel greases and wear reducers.

#### 4.1.3. Selection of Grease and Additives

**Figure 19:**
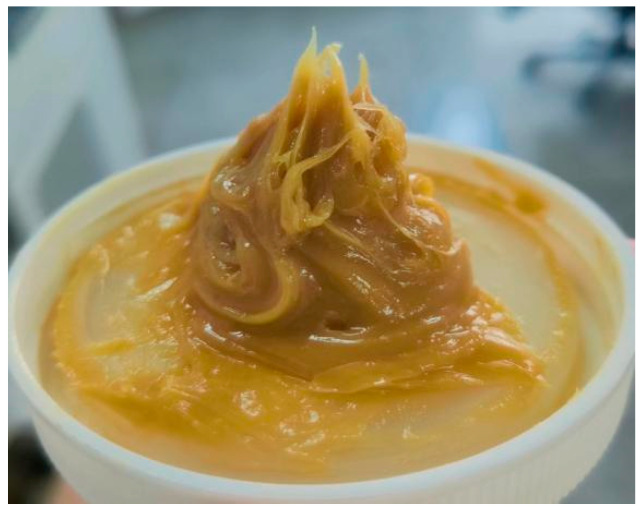
Schaeffler Load 460 gel grease.

Schaeffler Load 460 gel grease was selected as the experimental grease (as shown in Figure 19) (see Table 1 for detailed parameters). Schaeffler Load 460 grease is a high-performance, high-quality gel grease containing antioxidant and corrosion-resistant additives, which effectively protects the parts of the equipment from the effects of oxidation and corrosion, with excellent high-temperature performance and pressure resistance. It has excellent high temperature performance and pressure resistance. These properties are particularly important in high-end equipment, such as industrial machinery, the automotive industry, and other equipment requiring high temperature and high pressure working environments. The grease maintains stable lubrication at high temperatures and is not prone to failure at high pressures, ensuring reliable operation over long periods of time. Long-term stability and resistance to oxidation and corrosion make it the lubricant of choice for many high-end equipment.

RFM3000 and PV611 friction modifiers have been selected. RFM3000’s main ingredient is molybdenum dialkyl dithiocarbamate (MoDTC), which has good chemical stability. Molybdenum dialkyl dithiocarbamate is an excellent extreme-pressure additive that forms a protective film under extreme pressure to prevent contact and wear on metal surfaces. Used in conjunction with high viscosity greases, it can significantly improve the extreme-pressure performance of the grease, ensuring the safe operation of equipment under high loads and challenging operating conditions. The pv611 friction modifier is a fatty acid friction reducer produced by Lubrizol, USA, which has been rated by a variety of certifications. Polysiloxanes have good high temperature stability and antioxidant properties, which can help the grease to maintain stable lubrication performance in high-temperature environments, reducing the need for greases to be used at higher temperatures and reducing the need for lubricants to be used at lower temperatures. Polysiloxanes have good high temperature stability and oxidation resistance, which can help grease maintain stable lubricating performance in high-temperature environments and reduce grease loss and degradation. Increasing the viscosity of the grease will help to maintain effective lubrication at high temperatures for a longer period of time.

PV611 and RFM3000, two kinds of wear-reducing agents, are selected in the ratio of (3:1,1:1, and 1:3), respectively. At the (3:1) ratio, PV611 accounts for a higher proportion and is mainly used to enhance the viscosity and stability of the grease. The dominant role of PV611 is more significant at this ratio. The amount of RFM3000 is relatively small, and its contribution to friction reduction and anti-wear performance can be observed at a low concentration. This ratio can help to study the fundamental role of PV611 on lubrication performance while evaluating the auxiliary role of RFM3000. The ratio of (1:1) provides a balanced formulation of the two additives and helps to systematically study their synergistic effects. At this ratio, the effects of PV611 and RFM3000 will be more balanced and their combined effect can be better evaluated. The higher concentration of RFM3000 in the (1:3) ratio means that its friction reduction and the anti-wear properties of the grease will be dominant. The low percentage of PV611 allows the study of whether its viscosity-modifying effect is still effective under these conditions and the main effect of RFM3000. Such ratios can be useful in exploring how friction reduction and antiwear properties can be improved whilst maintaining the other properties of the grease at lower PV611 concentrations.

### 4.2. Experimental Program Design

#### 4.2.1. Tribological Experimental Design

**Figure 20:**
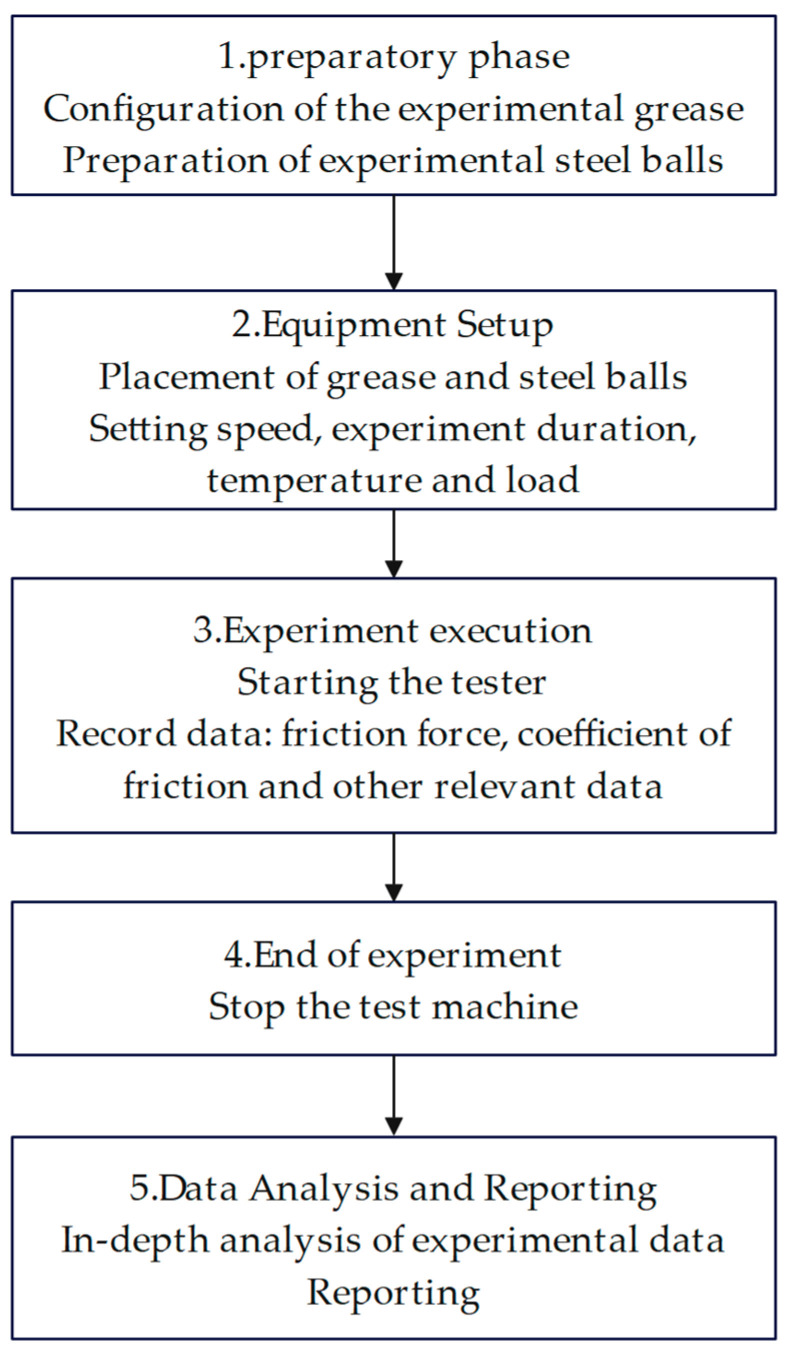
Flowchart of tribological experiments.

In this study, two additive blends of PV611 and RFM3000 (3:1, 1:1, and 1:3) were added to Schaeffler Load 460 grease by using a crossover experiment. Under the above conditions, the initial grease was prepared with the same proportion of additives (0.1%) as the experimental grease and the experimental steel ball, which should be smooth and clean to ensure the accuracy of the experimental results. Ensure that the grease and ball are placed in the proper position on the testing machine. Adjust the machine settings, including speed, duration, temperature, and load, according to the test requirements. Start the tester and begin the experiment. During the test, accurately record key data such as friction force, coefficient of friction, and ball wear. When the test is complete, stop the testing machine. Through the in-depth analysis of the test data, draw conclusions and form a report to evaluate the performance of the grease under actual working conditions. The flow chart of the tribological experiment is shown in Figure 20, and the experimental conditions of the equipment are shown in Table 2.

#### 4.2.2. Rheological Experimental Design

**Figure 21:**
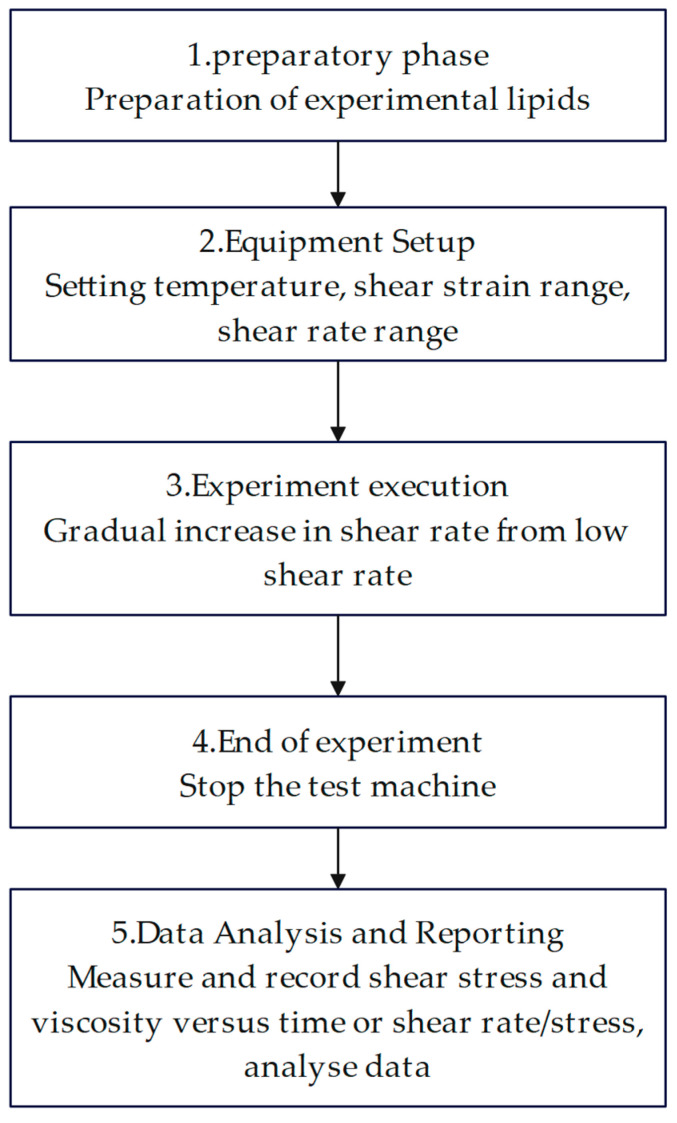
Flowchart of rheological experiment.

Since the standard operating temperature range of large high-end equipment is usually between −40 °C and 85 °C, and the operating temperature of the bearing during operation does not exceed 80 °C, the temperature measurement point selected in this paper is 80 °C. The shear strain range is 0.1% to 100%, and the controlled shear rate range is 0.01 s^−1^ to 100 s^−1^ for viscous shear experiments, etc. The prepared grease samples are put into the measurement system of the rheometer to ensure that the samples are in good contact with the sensors and there are no air bubbles. The rheometer applies different shear rates or shear stresses and records the response of the sample. Typically, it starts with a low shear rate or stress and gradually increases to a higher value. The rheometer records a real-time curve of shear stress and viscosity over time or shear rate/stress. The flow chart of the rheological experiment is shown in Figure 21, and the experimental conditions of the equipment are shown in Table 3.

τ=η·γ

τ is the shear stress (in Pa or N/m^2^)

η is the viscosity (in Pa·s or N·s/m^2^)

γ is the shear rate (in 1/s)

**Table 3:** Rheological experimental equipment conditions.

Test Methods	Test Parameters
Equipment model	MCR Rotational Rheometer
Shear strain range	0.1%~100%
Shear rate range	0.01 s^−1^~100 s^−1^
Temp	80 °C
Timing	15 s

## Figures and Tables

**Table 1:** Parameters related to Schaeffler Load 460 gel grease.

Grease Grades	Base Oil Viscosity	NLGI Consistency Grade	Operating Temperature	Wear Resistance	Antioxidant Properties	Corrosion Resistance
Schaeffler Load 460	400	1	−40 °C~130 °C	High	High	High

**Table 2:** Conditions of experimental equipment for tribology.

Test Methods	Test Parameters
Equipment model	MRS-10G Lever Type Four-Ball Friction Tester
Spindle speed	1200 r/min
Temp	80 °C
Payloads	392 N
Timing	3600 s

## Data Availability

The data presented in this study are openly available in article.
